# PARTICIPATION IN VALUED ACTIVITIES, RESTRICTION, AND SOCIAL ISOLATION IN DEMENTIA CAREGIVING

**DOI:** 10.1093/geroni/igae098.3681

**Published:** 2024-12-31

**Authors:** Sarah Won, Valerie Cotter, Chakra Budhathoki, Natalie Regier

**Affiliations:** Johns Hopkins University School of Nursing, Baltimore, Maryland, United States; Johns Hopkins University School of Nursing, Johns Hopkins School ofNursing, Maryland, United States; Johns Hopkins University, Baltimore, Maryland, United States; Johns Hopkins University School of Nursing, Baltimore, Maryland, United States

## Abstract

This study investigated the associations between five indicators of social determinants of health (SDOH) and participation in valued activities, participation restriction, and social isolation among family and other unpaid caregivers of older adults with dementia. Data from the 2022 National Health and Aging Trends Study and National Study of Caregiving IV (Round 12) were analyzed. The sample comprised 757 caregivers of community-dwelling care recipients aged 65 and older with possible/probable dementia. SDOH indicators included household income, education level, health insurance coverage, and neighborhood characteristics (neighborhood physical disorder and social cohesion of care recipients). Multivariable logistic regression models examined associations between SDOH indicators and caregiver outcomes, adjusting for demographics (race/ethnicity, age, gender, and marital status), health conditions, and caregiving characteristics (relationship with the care recipient, duration of caregiving year, hours helped last month, and informal/formal social support). Higher household annual income was significantly associated with increased odds of participation in valued activities (OR=3.46 for $30,000-$70,000, OR=3.40 for $70,000+, p< 0.01) and decreased odds of social isolation (OR=0.19 for $30,000-$70,000, OR=0.26 for $70,000+, p< 0.05) compared to income <$30,000. Higher education level (associate’s degree or above) was associated with significantly higher odds of participation in valued activities (OR=6.17, p< 0.001) compared to high school or less. No significant associations were found between SDOH indicators and participation restriction. Household income and education level were crucial SDOH factors associated with participation and social isolation. These findings highlight the importance of considering SDOH factors in developing interventions and policies to support dementia caregiving.

